# In silico analysis of driver genes in squamous cell carcinoma of the cervix: insights into their biological functions, prognosis, immune infiltration, and therapy

**DOI:** 10.1186/s12957-026-04435-y

**Published:** 2026-06-01

**Authors:** Samatha Bhat, Vasudha Devi, Shama Prasada Kabekkodu

**Affiliations:** 1 Department of Biotherapeutics Research, Manipal Academy of Higher Education, Manipal, India; 2 Department of Basic Medical Sciences, Manipal Academy of Higher Education, Manipal, India; 3 Department of Cell and Molecular Biology, Manipal School of Life Sciences, Manipal Academy of Higher Education, Manipal, India

**Keywords:** Cervical cancer, In silico analysis, Driver genes, Mutations, Hub genes, Prognostic marker, Immune infiltration

## Abstract

**Background:**

Cervical cancer is the fourth most common cancer affecting the female reproductive system worldwide. Although several studies have reported recurrent mutations in cervical squamous cell carcinoma (SCC), a comprehensive understanding of the clinically relevant driver genes remains limited. Unlike previous single-cohort or frequency-based reports, this study integrates four independent cervical squamous cell carcinoma (SCC) cohorts with network-based analysis, multiendpoint survival assessment, and drug–gene interaction to prioritize functionally and clinically relevant driver hub genes.

**Materials and methods:**

In this study, we performed a genomic analysis of a cohort of 4 squamous cell carcinomas of the cervix (SCCs), consisting of 467 samples, to identify driver genes and their clinical significance. Key pathways and biological functions affected were tested by functional enrichment analysis. The Search Tool for the Retrieval of Interacting Genes/Proteins (STRING) and CytoHubba tools were used to construct a protein‒protein interaction network (PPIN) and identify the hub genes. Additional analyses included enrichment assessment of cancer hallmarks, survival evaluation, immune cell infiltration profiling, and drug‒gene interaction studies.

**Results:**

Our analysis revealed 9,749,109 mutations across 44 driver genes. *PIK3CA*,* KMT2C*,* KMT2D*,* FBXW7*,* FAT1*,* EP300*,* TP53*,* NOTCH1*,* STK11*, and *CASP8* were the top 10 mutated genes. *PIK3CA*,* NOTCH1*,* PTEN*,* KRAS*,* ERBB2*,* TP53*,* ARID1A*,* EP300*,* STK11*, and *FBXW7* emerged as the top 10 hub genes according to the results of the PPIN and Cytohubba analyses. In addition, we observed significant differences in T helper cell type 2, natural killer cell, dendritic cell, and gamma delta T-cell composition in samples with hub gene mutations. Prioritization analysis of drug and hub gene interactions revealed 112 clinically relevant compounds, especially HER2-directed therapies (trastuzumab), PI3K inhibitors (alpelisib), and mTOR inhibitors (everolimus).

**Conclusion:**

Collectively, our analysis describes the driver genes and mutation characteristics in SCC. The multi-cohort and network-based framework employed in this study identifies candidate hub genes that warrant further clinical investigation in cervical cancer.

**Supplementary Information:**

The online version contains supplementary material available at 10.1186/s12957-026-04435-y.

## Introduction

Cervical cancer is the predominant cancer affecting females in several low- and middle-income countries [1]. Globally, cervical cancer ranks as the fourth most prevalent gynecological malignancy, with 660,000 cases and 350,000 cases in 2022 [2]. In India, 123,907 new cases and 77,348 deaths due to cervical cancer have been reported [3]. HPV vaccination and the effective implementation of cervical screening programs have helped to significantly reduce the cervical cancer burden globally. However, because of socioeconomic factors, cervical cancer is mostly detected at an advanced stage in several countries [4]. The current standard of care aimed at improving cervical cancer patient survival is the use of neoadjuvant chemotherapy followed by radical surgery or concurrent chemoradiotherapy. One study highlighted the heterogeneity in the overall survival rate among patients with stage IB2–IIIB disease using survival nomogram-based analyses after neoadjuvant chemotherapy and surgery [5]. Additionally, the addition of immune checkpoint inhibitors to standard chemoradiotherapy regimens has improved overall survival [6]. Management of advanced cervical carcinoma is often challenging, with many patients experiencing poor clinical outcomes, including lymph node metastasis, relapse, acquired treatment resistance, and reduced overall survival [7]. Thus, identifying molecular targets that may improve patient care is important.

Infection with high-risk HPV and genetic changes have been shown to play crucial roles in cervical cancer [8]. Genome-wide studies in cervical cancer have indicated the occurrence of widespread genomic and epigenomic alterations [9]. For example, a study by Chen et al. revealed associations between *DAP*,* NR5A2*, and *MIR365-2* and the development of cervical cancer [10]. Another study reported recurrent somatic mutations in *MAPK1* in cervical squamous cell carcinoma patients [11]. Genome-wide analyses of the TCGA-CESC dataset revealed TTN, PIK3CA, MUC4, KMT2C, MUC16, KMT2D, SYNE1, FLG, DST, and EP300 as the most frequently mutated genes in cervical carcinoma [12]. Cervical carcinoma with *PIK3CA* and *EGFR* amplification and activating mutations in *PIK3CA* and *KRAS* correlated with poor outcomes. A reanalysis of the TCGA-CESC dataset by Xu and colleagues revealed ten possible driver genes in cervical cancer, including GPR107, CHRNA5, Rb1, and H2BFM [12].

Driver mutations are a subset of somatic mutations with the potential to initiate and promote cancer [13]. Driver mutations can influence patient survival, recurrence and therapeutic response in patients with cervical cancer. Evaluation of 284 cervical cancer specimens in the TCGA-CESC dataset revealed driver genes that could serve as biomarkers for molecular categorization. However, this study only assessed the potential of driver mutations within the TCGA-CESC dataset and did not include external validation. Thus, additional analyses using multiple datasets are needed to identify the most prevalent driver mutations to understand their clinical importance. Previous driver gene analysis depended solely on TCGA-CESC datasets and mutation frequency. Compared with previously published gene analyses, the present study has several novelties. First, we analyzed four independent cervical cancer cohorts comprising 467 samples to identify the most common driver genes. Second, we conducted a protein‒protein interaction network (PPIN) analysis and used statistical analysis to select key genes (hub genes) instead of just ranking them by mutation count. Third, we systematically compared the mutation status of the hub genes with multiple survival outcomes, such as overall survival (OS), progression-free survival (PFS), disease-specific survival (DSS), and relapse-free survival (RFS). Fourth, the associations between hub gene mutations and immune infiltration patterns were compared. Finally, we analyzed the impact of mutation on hub gene expression, performed clinical covariance analysis, and validated protein expression via immunohistochemistry. Collectively, this integrative framework moves beyond a list of mutated genes to provide a set of clinically relevant genes.

## Methodology

### Data resource and driver gene identification

IntOGen (https://www.intogen.org/search) is a cancer genomics framework for the extraction and analysis of mutation data from tumor samples generated from genome-wide studies to identify driver genes [14]. IntOGen tools integrate genomic and clinical information from 73 cancer types, 33,019 samples, 252,486,809 mutations, and 619 driver genes generated from WXS and WGS datasets. To reduce heterogeneity arising from combining WXS and WGS datasets, IntOGen applies a rigorous preprocessing step that removes hypermutated samples, uncertain variants, and mutations in low-mappability regions. Furthermore, the data generated from two different platforms (WXS and WGS) were not combined or analyzed together. Instead, the data generated from each platform were analyzed separately, and the mutated genes identified were subjected to driver gene prediction using IntOGen’s driver gene prediction pipelines and are presented as supplementary tables. Consequently, the pipeline outputs gene-level driver calls rather than quantitative allele frequency estimates, which are more robust to batch effects across platforms. Genes whose somatic mutations confer a selective growth advantage and causally contribute to tumorigenesis are considered driver genes. The driver genes were identified based on the IntOGen mutation pipeline. Genes with a high mutation frequency, regardless of functional impact, were defined as frequently mutated genes. First, it assesses the functional impact of somatic mutations using tools such as SIFT, PolyPhen2, and MutationAssessor, optionally applying an expression filter to exclude nonexpressed genes. It then employs two complementary, reimplemented methods: (i) OncodriveFM, which evaluates whether a gene shows a bias toward the accumulation of mutations with high functional impact compared with the background distribution, and (ii) OncodriveCLUST, which assesses whether protein-affecting mutations are significantly clustered along protein sequences relative to silent mutations. The candidate driver genes and pathways are predicted from the p-values and false discovery rates generated through these analyses.

In this study, we analyzed the mutation profiles and associated clinicopathological information of patients with squamous cell carcinoma of the cervix (SCC) [*n* = 467: TCGA_WXS_CESC (*n* = 288), CGCI_WGS_CESC_2020 (*n* = 120), HARTWIG_WGS_CESC_2020 (*n* = 41), and PCAWG_WGS_CERVIX_SCC (*n* = 18)] to identify and visualize potential driver genes via IntOGen. The IntOGen web server was queried with the search term “Cervical Squamous Cell Carcinoma” to identify potential driver genes. The output generated from the querying IntOgen tool was directly used for downstream analysis.

### Mutation analysis

OncoVar (https://oncovar.org/) tools were used to systematically prioritize and visualize the mutated genes in cervical cancer [15]. The tool uses previously published algorithms and integrates driver events with clinical data to identify and visualize driver genes and driver mutations generated from analyses of The Cancer Genome Atlas (TCGA) and International Cancer Genome Consortium (ICGC). The OncoVar tool predicts the pathogenicity of variation in ICGA data, specifically focusing on missense mutations using REVEL and VEST3, filtering variants through gnomAD, and incorporating AI-Driver prediction as an additional covariate. TCGA data were analyzed for all mutations using statistical tools (MutSigCV, Fisher’s exact test, and MutPanning) to identify significantly mutated genes. Databases such as OncoKB and FASMIC were used to predict extreme mutations and subsequently classify driver mutations. All the analyses were carried out using the default parameter set in the OncoVar tool. The OncoVar tool contains information on 20,162 cancer driver mutations distributed in 814 driver genes analyzed from 33 to 18 different cancer types from the TCGA and ICGA, respectively.

### Pathways and GO enrichment analysis

The Kyoto Encyclopedia of Genes and Genomes database was used to identify potential associations of genes with pathways, chemical compounds, drugs, and diseases (https://www.genome.jp/kegg/). Gene Ontology (GO) enrichment analysis was used to annotate the associations of genes with gene products, which included biological processes (BP), cellular components (CC), and molecular functions (MF). The OncoVar tool was used to identify driver pathways and GO processes [15]. OncoVar integrates driver gene information with that of KEGG and GO to identify potential pathways and GO processes that may drive carcinogenesis. GO enrichment is performed via a hypergeometric test integrated within the OncoVar tool to assess pathway involvement and construct driver networks. A P value less than 0.05 was considered to indicate statistical significance.

### PPIN construction, hub gene identification and visualization

A protein‒protein interaction network (PPIN) was constructed to investigate the functional interactions of putative driver genes via the Search Tool for the Retrieval of Interacting Genes (STRING) V12.0 (https://string-db.org/) [16] online tool and visualized via Cytoscape 3.10.2 (https://cytoscape.org/download.html) [17]. The list of candidate driver genes identified from the previous steps was used as the seed set. The network was constructed using a confidence score of ≥ 0.7 to minimize false positives. The top ten genes with the greatest connections (hub genes) were identified using CytoHubba (https://apps.cytoscape.org/apps/cytohubba) [18]. The top 10 nodes were ranked by the maximal clique centrality (MCC) method integrated into the CytoHubba tool. Genes occupying central topological positions in the PPIN identified using the CytoHubba tool via the MCC algorithm were considered hub genes. A P value less than 0.05 was considered to indicate statistical significance. Gene set enrichment analysis for cancer hallmarks was carried out using the CancerHallmarks.com tool [19].

### Survival analysis

To assess the relationship between hub driver gene mutations and survival, the Tumor Online Prognostic Analyses Platform (ToPP) was used [20]. Univariate and multivariate analyses were carried out to determine hazard ratios (HRs) to estimate the impact of mutations on patient survival. We used the TCGA-CESC dataset, best cutoff, 95% confidence interval and mutation information as parameters to construct Kaplan–Meier survival plots via the ToPP web server. Patients from the TCGA-CESC dataset were stratified into two groups based on their mutation status of the top 10 hub genes: the high-risk group (samples harboring mutations in one or more hub genes) and the low-risk group (samples with no mutations in any of the 10 hub genes). The optimal cutoff for stratification was determined using the platform’s default algorithm to maximize survival discrimination. Kaplan–Meier survival curves were generated to compare overall survival (OS), progression-free survival (PFS), disease-specific survival (DSS), and relapse-free survival (RFS) between the two groups. Univariate and multivariate Cox proportional hazards models were utilized to identify hazard ratios (HRs) with 95% confidence intervals (CIs).

### Immune infiltration analysis

The Gene Set Cancer Analysis (GSCA) webserver was used for immunogenomic analyses [21]. The ImmuCellAI algorithm combines RNA-seq and microarray data to calculate the enrichment of 22 distinct types of immune cells. The CIBERSORT algorithm was employed on the TCGA bulk RNA-seq data to determine the relative abundance of 22 immune cell types. Moreover, single-sample gene set enrichment analysis (ssGSEA) integrated in the GSVA package was used to calculate enrichment scores for immune-related gene signatures. Differential immune infiltration between the high and low driver gene expression groups (split by median) was tested using the Mann‒Whitney U test, and correlations were assessed via Spearman’s rank correlation (adjusted *p* < 0.05). All immune infiltration analyses are predictive and correlational; no causal inference is made between mutation status and immune cell abundance.

### Drug–gene interaction analysis

The webserver PanDrugs2 was used to predict potential drugs that target driver hub genes [22] (https://www.pandrugs.org/#!/) using the default parameters. PandDrugs is a bioinformatic pipeline that prioritizes drugs on the basis of multi-OMICS data. The PanDrug webserver integrates drug target associations generated from 4642 genes and 14,856 compounds. The initial PanDrugs2 output (410 compounds) was refined to identify clinically actionable drugs targeting hub genes. Drugs were prioritized based on (a) FDA approval for any cancer indication, (b) inclusion in NCCN guidelines for cervical or gynecologic cancers, or (c) active phase 2/3 clinical trial evaluation in cervical cancer (ClinicalTrials.gov). The candidates with D scores > 0.7 and G scores > 0.6 were considered the best therapeutic candidates according to PanDrugs2 confidence metrics.

### Gene expression and clinical covariate analysis

The online tool OncoDB 2.0 (https://oncodb.org/index.html) was used to test the potential association of mutations in hub genes with linked gene expression and clinical parameters. The tool consists of multi-OMICS data from 10,000 patients across 33 cancer types. This tool allows comparisons of mutation profiles linked to clinical parameters. The TCGA WGS datasets were preprocessed using the variant-calling pipeline utilized in the IMAPR project. Briefly, whole-genome reads were mapped using BWA-MEM, after which PCR duplicates were removed using GATK, followed by recalibration using BaseRecalibrator and ApplyBQSR. The somatic variants were called GATK MuTect2. For the RNA-seq data analysis, the raw reads from tumor and normal samples were compared with the human genome by alignment using the STAR tool. The aligned reads were mapped to the human RefSeq transcriptome, followed by raw read normalization using the transcripts per million (TPM) method. The differences in the expression of hub genes between mutated and nonmutated samples were compared using the mutational expression profile option within the OncoDB 2.0 tool. The statistical significance of differential expression was estimated between mutated and non-mutated genes via Student’s *t-test*. The associations of mutations in hub genes with clinical parameters such as clinical stage, histological grade, and pathological stage were evaluated using the somatic mutation and mutation clinical profile options of the OncoDB 2.0 tool. Fisher’s exact test was used to determine significant differences between the groups.

### IHC analysis using the human protein atlas

Immunohistochemistry (IHC) data for the selected hub genes were retrieved from the Human Protein Atlas (HPA; https://www.proteinatlas.org/). The HPA provides prevalidated IHC data for a wide range of tumor tissues. The expression levels of proteins were categorized as high, medium, low, or not detected on the basis of the antibody staining intensity and fraction of positive cells. The protein expression of each hub gene was determined for patients with squamous cell carcinoma of the cervix (*n* = 12). Expression was classified as “high or medium” (clinically relevant overexpression) or “low or no expression” (loss or minimal expression), as per the HPA semiquantitative scoring guidelines.

### Statistical analysis

All the statistical analyses were performed using the built-in modules of the respective web-based tools (IntOGen, OncoVar, ToPP, GSCA, and PanDrugs2) and the Cytoscape software environment. For multiple comparisons, p values were adjusted using Benjamini–Hochberg false discovery rate (FDR) correction wherever applicable. A two-sided p value < 0.05 was considered to indicate statistical significance.

## Results

### Molecular analysis strategies

**Fig. 1:**
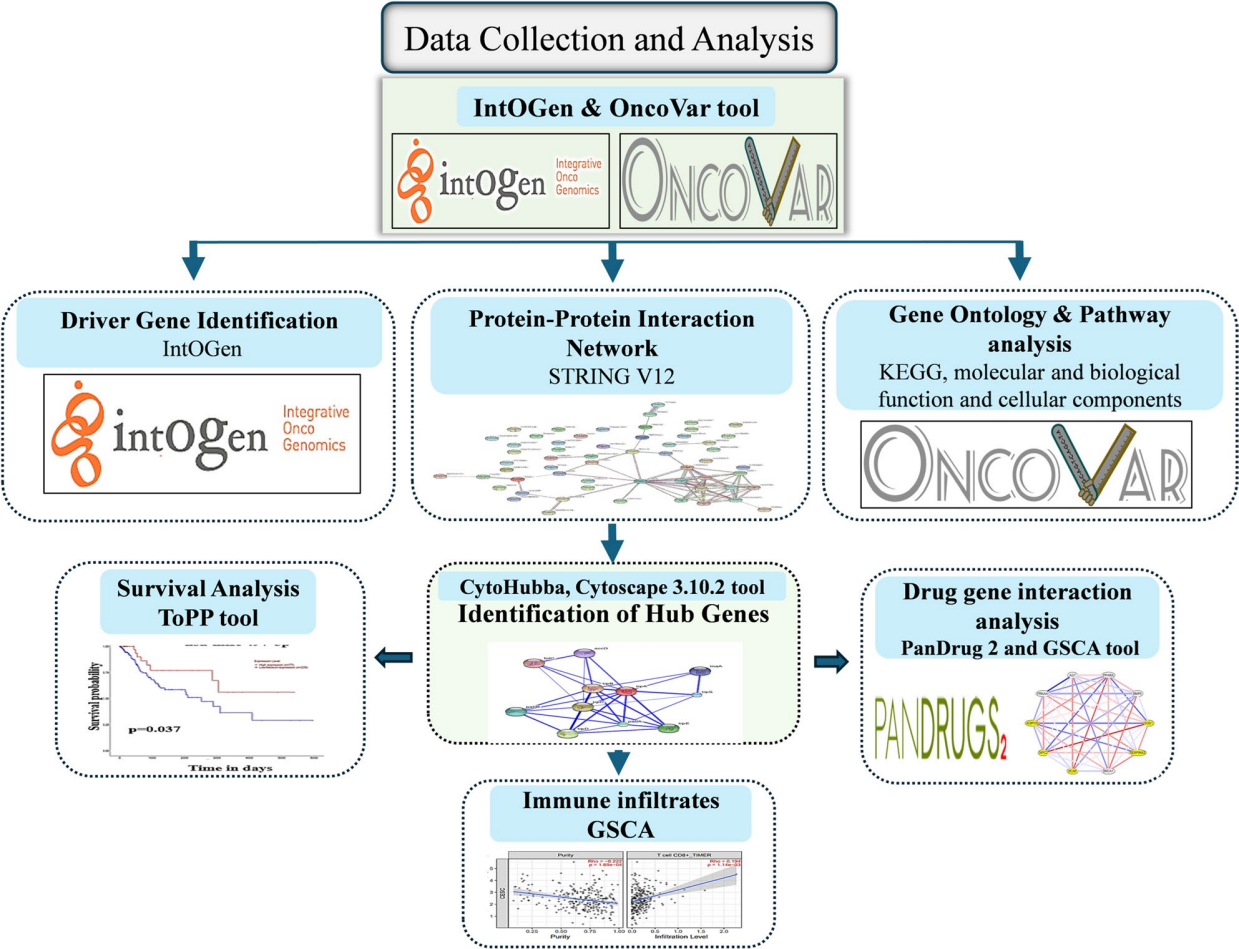
Schematic diagram representing the overall approach adopted in the present study to identify potential driver genes and their role and clinical significance in SCC

We systematically analyzed SCC datasets and identified driver genes and mutations, pathways affected, and potential druggable genes and novel drugs that can be used in SCC patients via publicly available genomics data. The potential driver genes and mutations were first identified via the IntOgen and OncoVar web servers. The protein interaction network of driver genes created by STRING was analyzed using cytoHubba in Cytoscape. This process helped identify the ten most important hub genes on the basis of their topology. Functional and pathway enrichment analyses of the top 10 hub genes were performed using the OncoVar tool. Univariate and multivariate analyses were performed via the ToPP online tool to assess the associations of mutations with the survival of patients with cervical cancer. The ImmuCellAI algorithm integrated with the GSCA tool was used to determine the associations between driver gene mutations and immune cell infiltration. We employed PanDrug 2 and STITCH (http://stitch.embl.de/cgi/about.pl) platforms to identify and prioritize pharmacologically actionable compounds targeting the identified molecular vulnerabilities in cervical cancer (Fig. 1).

### Patient cohorts

Our analysis included 467 samples from 4 independent cohorts of SCC samples, such as Cervical Squamous Cell Carcinoma from TCGA/PanCatAtlas, Phs000178 (TCGA_WXS_CESC, *n* = 288; driver genes: 33; mutations: 103,550), Cervical Squamous Cell Carcinoma from CGCI (CGCI_WGS_CESC_2020; *n* = 120; driver genes: 17; mutations: 8,674,181), Cervical Squamous Cell Carcinoma from Hartwig Medical Foundation (HARTWIG_WGS_CESC_2020; *n* = 41; driver genes: 17; mutations: 850,296), and Cervical Squamous Cell Carcinoma from PCAWG (PCAWG_WGS_CERVIX_SCC; *n* = 18; driver gene: 4; mutations: 121,082).

### Mutations in cervical cancer samples

**Fig. 2:**
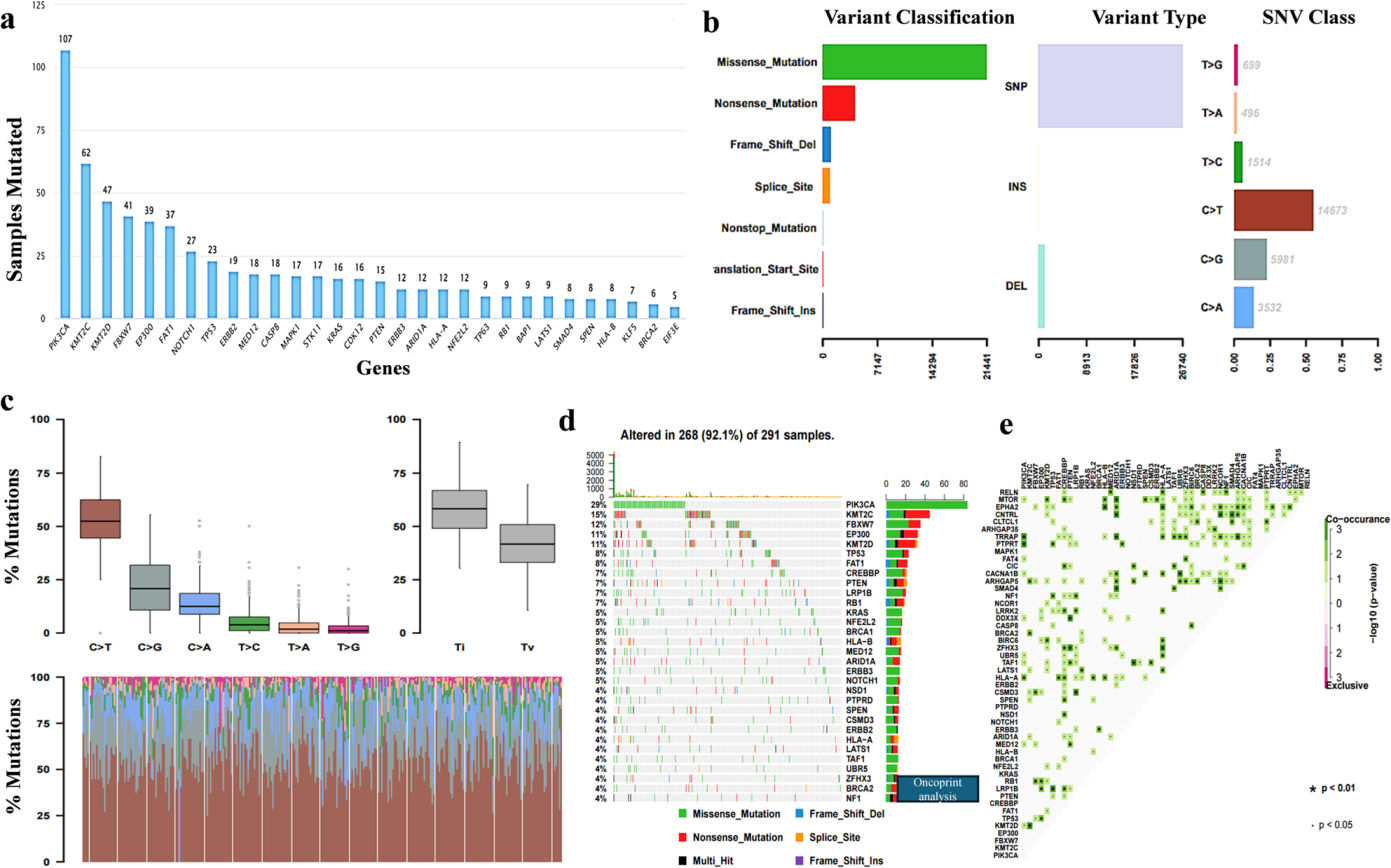
Overview of driver genes and mutations in SCC of the cervix. **a** Bar graph showing the most recurrently mutated driver genes in cervical cancer. The plot configuration features sample mutation status along the horizontal axis and corresponding gene identifiers on the vertical axis. **b** Overall gene mutation summary plots. Missense mutations were the most frequently observed genomic alterations in SCC patients. **c** Transition and transversion summary plot. C > T change was the most frequent change observed in patients with SCC of the cervix. **d** Waterfall plots for the top 30 mutated genes in patients with SCC of the cervix. **e** Interaction plots of the top mutated genes in patients with SCC of the cervix

Our analyses focused on gene-level mutation frequencies and recurrent alterations to minimize platform-specific biases. Analysis of cervical cancer samples via IntOGen revealed 44 driver genes (Supplementary Table 1). Among the 44 driver genes identified, *PIK3CA* and *STK11* were mutated in all four datasets. The mutation frequency of the driver genes ranged from 0.43% to 25.27% of the samples. *PIK3CA*,* KMT2C*,* KMT2D*,* FBXW7*,* FAT1*,* EP300*,* TP53*,* NOTCH1*,* STK11* and *CASP8* were the top ten mutated genes, with mutation frequencies ranging from 4.28% to 25.27% (Fig. 2 and Supplementary Table 1). Among the 44 driver genes, *PIK3CA* presented the greatest number of mutations (107) (Fig. 2a). The number of mutations in the top ten highly mutated driver genes ranged from 25 to 137. Among the various changes, the number of missense mutations was greatest, followed by the number of nonsense mutations (Fig. 2b). Single-nucleotide polymorphisms were the most common variant type (Fig. 2b). C > T change was the most frequent SNV class detected in cervical cancer patients (Fig. 2c). The frequencies and types of the top 30 genes altered are shown in Fig. 2d. Our analysis revealed several genes that were frequently comutated in cervical squamous cell carcinoma samples (Fig. 2d).

### Functional enrichment analysis of the driver genes revealed enrichment of human papillomavirus infection and viral carcinogenesis

**Fig. 3:**
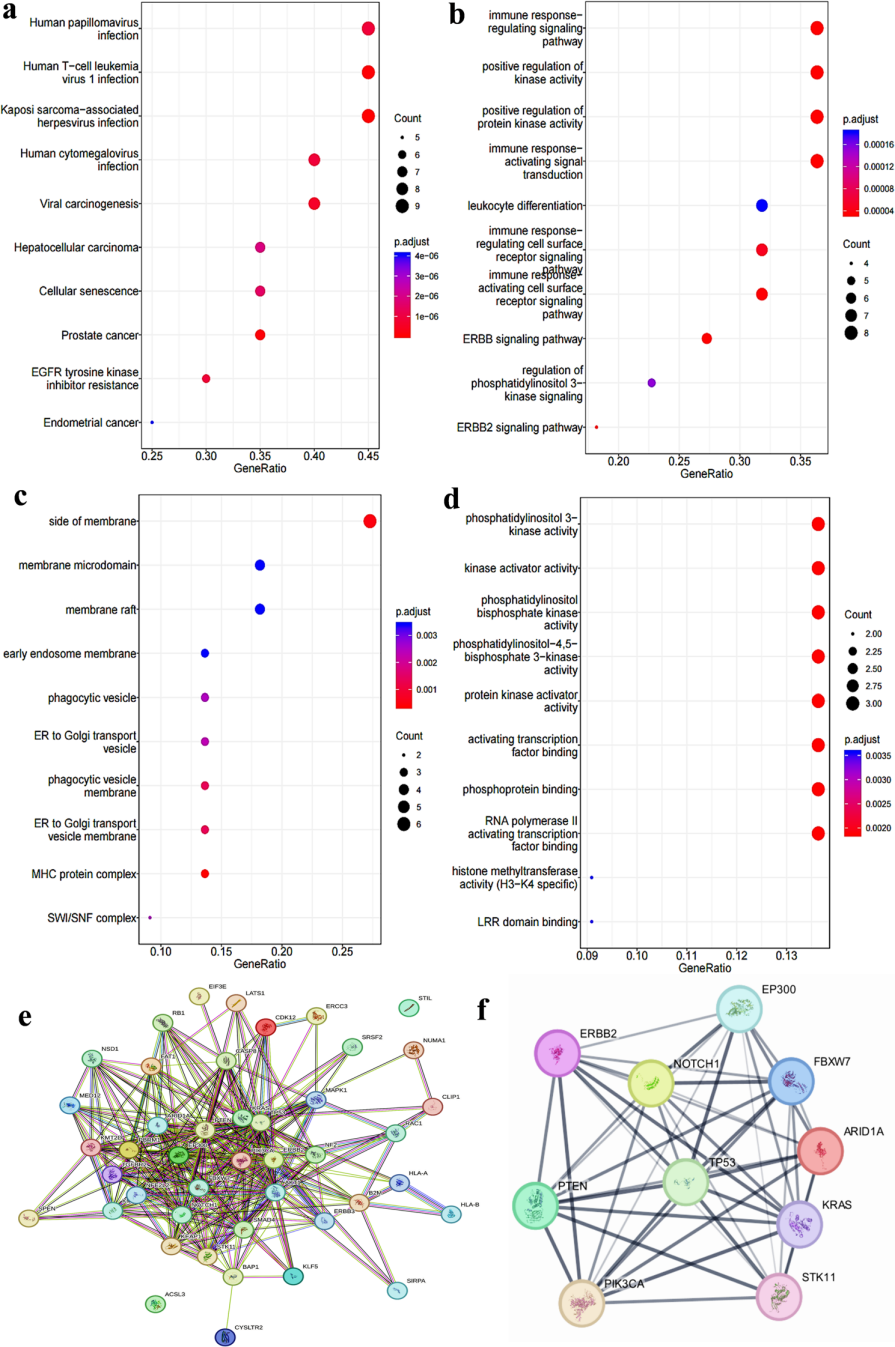
Functional enrichment analysis and PPIN of driver genes. **a** Dot plot representing the top ten enriched pathway terms compared with those in the KEGG database. **b** Dot plot analysis illustrating the predominant biological pathways enriched among the identified driver genes, highlighting the top ten processes. **c** Dot plot visualization of cellular component enrichment analysis for the identified driver genes. **d** Dot plot visualization of molecular function enrichment analysis for the identified driver genes. **e** PPIN showing the interactions between driver genes. **f** Interactions between the top ten driver genes

Pathway enrichment analysis of the 44 driver genes performed via OncoVar revealed enrichment of several oncogenic pathways. HPV infection, infection with various viruses, and viral carcinogenesis were the most enriched pathways. The top 10 pathways enriched in relation to 44 driver genes in cervical cancer are listed in Fig. 3a. The top ten enriched terms across the BP, CC, and MF categories for the SCC driver genes are systematically displayed in Fig. 3b, c and d. GO analysis suggested a possible association of 44 driver genes with immune response-regulating signaling, positive regulation of kinase activity, phosphatidylinositol 3 kinase activity, and others.

### PPIN and hub gene networks of driver genes

PPIN of 44 driver genes was generated via the STRING webserver to reveal the interactions among the driver genes (Fig. 3e). The constructed PPIN comprised 44 molecular nodes interconnected by 296 edges, demonstrating highly significant interaction enrichment (*p* = 1.0e-16). We carried out hub gene analysis using the cytoHubba tool to identify the most connected genes in the PPIN using the MCC method (Fig. 3f). Notably, hub status reflects network connectivity only and does not imply biological causality or functional importance. These genes are prioritized for downstream analysis based on their topological position, generating hypotheses for experimental testing. Hub gene analysis of 44 driver genes revealed that PIK3CA, NOTCH1, PTEN, KRAS, ERBB2, TP53, ARID1A, EP300, STK11, and FBXW7 were the top 10 proteins in the network (PPI enrichment p value of 1.47 e-09), suggesting their significant functional interactions in cancer-related protein networks.

### Hub genes and prognostic utility

**Fig. 4:**
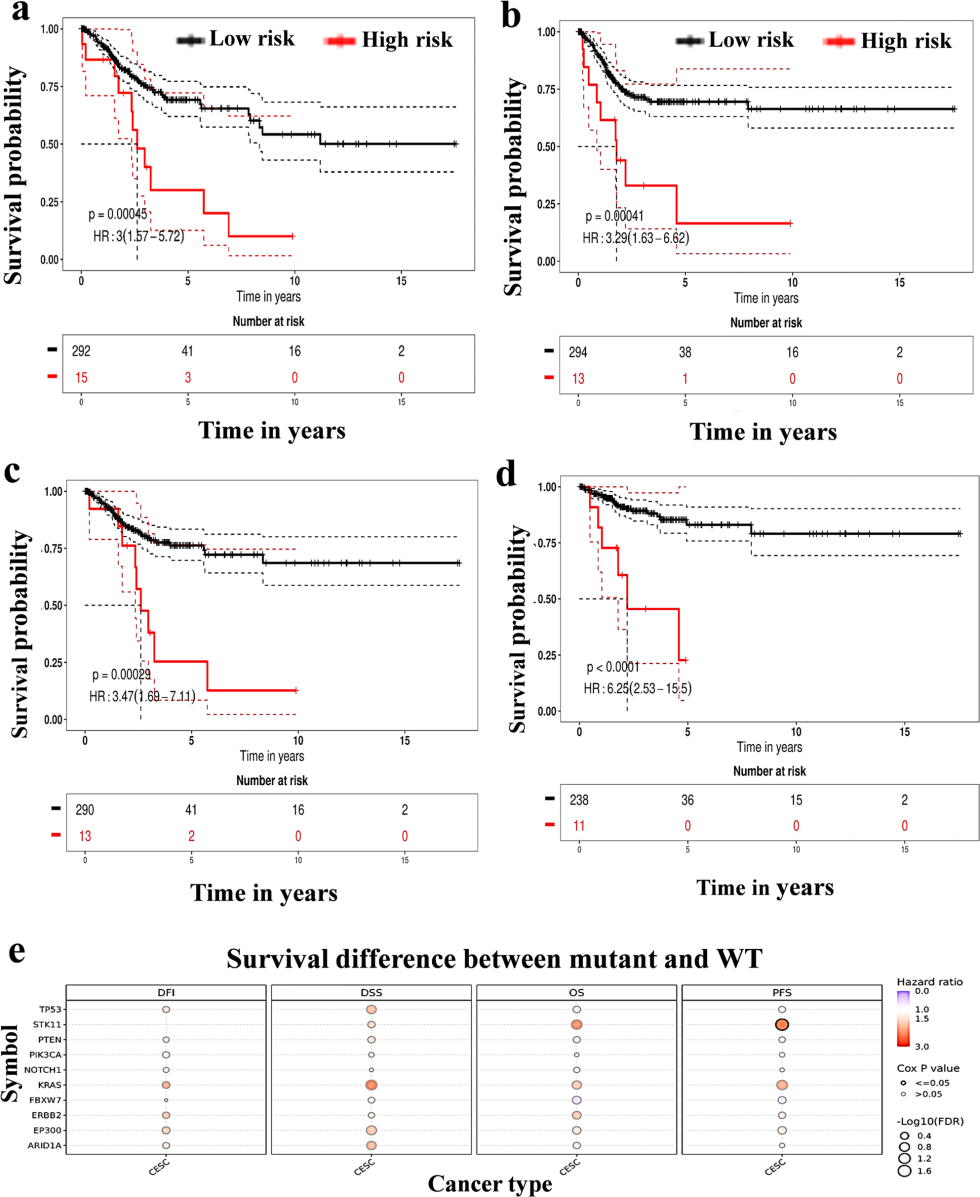
Driver hub gene mutations influence the survival of SCC patients. **a**-**d**) KM plot for overall survival, progression-free survival, disease-specific survival and relapse-free survival. Elevated risk scores were strongly inversely correlated with patient survival outcomes. Using the median risk score as the cutoff threshold, we stratified patients into distinct high-risk and low-risk prognostic categories. **e**). Differences in survival between mutant and wild-type samples at the individual gene level

Multivariate analysis was performed via the ToPP online tool to analyze the prognostic utility of mutated driver hub genes for the survival of patients with cervical cancer. The survival differences were compared between mutated samples and nonmutated samples, and the optimal grouping threshold was determined using software to maximize survival discrimination. Patients were stratified into two prognostic groups, wherein samples harboring mutations in one or more of the 10 identified driver hub genes were classified into the high-risk group, while samples without any mutations in these genes were categorized into the low-risk group. The high-risk group comprised a relatively small proportion of patients because of the relatively low frequency of mutations in the hub driver genes within the cohort. Multivariate analysis of all 10 driver hub genes revealed that mutations were associated with significantly poorer overall survival (HR: 3.0; *p* = 0.00045), progression-free survival (HR: 3.29; *p* = 0.00041), disease-specific survival (HR: 3.47; *p* = 0.00029), and relapse-free survival (HR: 6.25; *p* < 0.001) (Fig. 4a-e).

### Relationships between driver hub genes and the tumor immune microenvironment

**Fig. 5:**
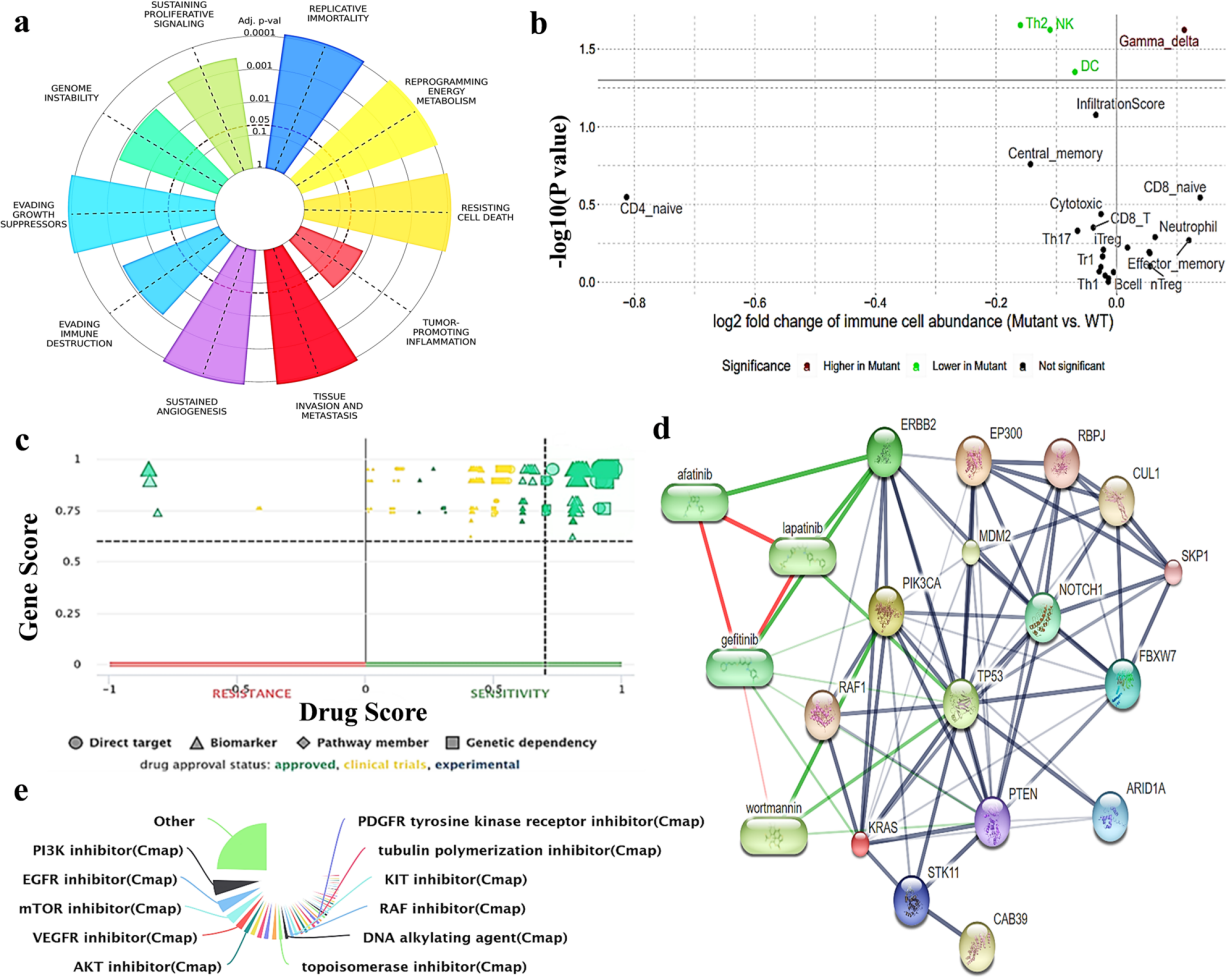
Immune infiltration and drug–gene interaction analysis: (**a**) Association between driver hub genes and cancer hallmarks. In silico analysis suggested that the top ten driver hub genes may promote the acquisition of various hallmarks of cancer. **b** Association between driver hub gene mutations and immune cell infiltration. **c** Scatter plot showing the drugs that are sensitive and resistant according to the hub gene profile. **d** Network demonstrating the interactions between drugs and driver hub genes. **e** Represents the drugs by family chart

To explore potential associations between genomic alterations and the tumor immune microenvironment, we performed a correlative analysis of driver hub gene mutation status and immune cell infiltration levels using the ImmuCellAI algorithm. Our analysis revealed that multiple hub genes are related to all the reported cancer hallmarks (Fig. 5a, Supplementary Table 2). We next compared the associations between driver hub gene mutations and 24 immune cell types via the ImmuCellAI algorithm integrated with the GSCA web tool. Mutations in 10 driver hub genes were associated with four immune cell types, namely, T helper 2 (TH2), natural killer (NK), dendritic (DC), and gamma delta cells (Fig. 5b, Supplementary Table 3).

### Prioritization of clinically actionable drug–gene interactions

The PanDrug2 tool was used to mine clinically relevant gene‒drug interactions. Analysis of the top 10 driver hub genes via PanDrug2 revealed 410 drugs (Supplementary Table 4) that were either in clinical trials or approved for treating cancers other than cervical cancer (Fig. 5c). Among the 10 genes queried, 8 genes (*ERBB2*,* KRAS*,* PIK3CA*,* PTEN*,* TP53*,* FBXW7*,* STK11*,* and NOTCH1*) exhibited drug–gene interactions (Fig. 5d). Inhibitors against PI3K, EGFR, mTOR, VEGFR, AKT, PDGFR tyrosine kinase, tubulin polymerization, KIT, topoisomerase, and RAF emerged as key drug families against the hub genes (Fig. 5e).

We filtered the data to prioritize clinically actionable interactions. After clinical prioritization filters (FDA approval for cancer, NCCN guideline inclusion, or active cervical cancer clinical trials; exclusion of noncancer drugs and dietary supplements) were applied, 112 clinically relevant compounds were retained (Supplementary Table 5). The complete list of 112 clinically prioritized drugs with individual gene targets, approval status, D score, G score and clinical evidence of cervical cancer is provided in Supplementary Table 5. We identified 29 FDA-approved drugs with established or emerging evidence in cancer. The list comprises agents such as HER2-directed therapies (trastuzumab, pertuzumab, lapatinib, neratinib, and tucatinib), agents targeting *ERBB2* alterations, PI3K inhibitors (alpelisib and copanlisib) for *PIK3CA* mutation, and mTOR inhibitors (everolimus and temsirolimus). In addition, we identified standard chemotherapy agents (cisplatin, carboplatin, paclitaxel, docetaxel, gemcitabine, and 5-fluorouracil), immunotherapy agents (pembrolizumab and bevacizumab) and PARP inhibitors (olaparib, niraparib, rucaparib, and talazoparib).

### Relationships between mutations and gene expression of hub genes

Analysis of mutation frequencies across clinical and pathological parameters revealed statistically significant associations for three genes (Supplementary Table 6). PIK3CA mutations were significantly associated with tumor grade (Fisher’s exact *p* = 0.01), with higher mutation frequencies observed in low-grade tumors (G1: 40.0%, G2: 39.8%) than in G3 (21.1%). ERBB2 mutation was significantly associated with nodal status (*p* = 0.037) and was present in N0 (6.1%) and N1 (8.9%) tumors but absent in all NX samples (0.0%). STK11 mutation was significantly associated with T stage (*p* = 0.016) and markedly enriched in T4 tumors (22.2%) compared with earlier stages (T1: 1.7%, T2: 4.3%, T3: 0.0%). Non-significant trends were observed for *TP53*,* EP300 and FBXW7*, wherein *TP53* mutations increased with advancing stage (Stage I: 6.4% to Stage IV: 15.0%) and grade (G1: 0% to G3: 13.8%). *EP300* mutations were more frequent in metastatic (M1: 30.0% vs. M0: 8.3%) and node-positive (N1: 16.1% vs. N0: 8.8%) tumors. The *FBXW7* mutations were more common in non-metastatic tumors (M0: 17.4% vs. M1: 0.0%).

**Fig. 6:**
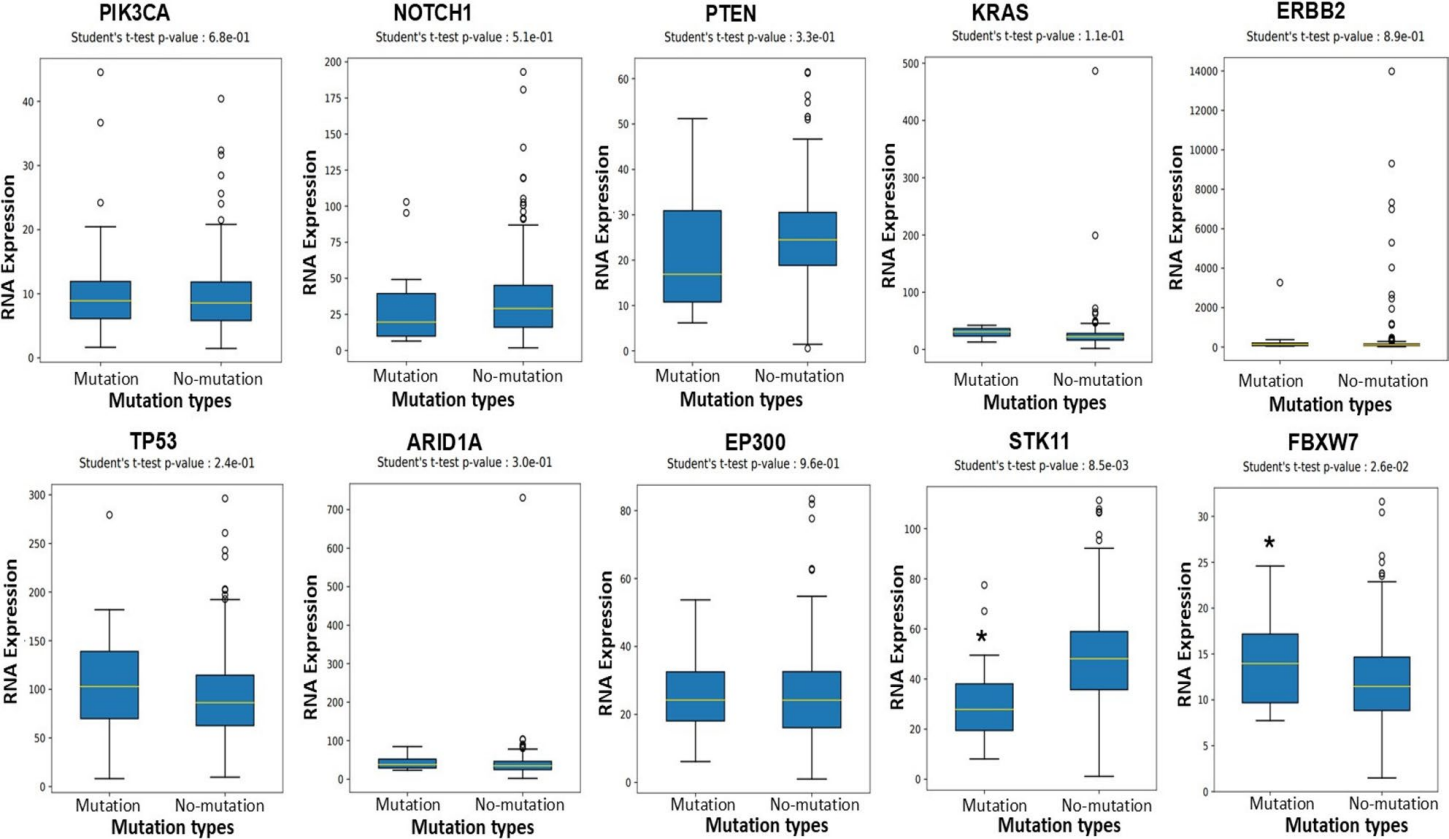
Association between mutation status and gene expression levels of driver genes in cervical squamous cell carcinoma. The figure illustrates the relationship between mutation status and gene expression for 9 driver genes. No statistically significant expression differences were observed between mutated and non-mutated groups for *PIK3CA*,* NOTCH1*,* PTEN*,* KRAS*,* ERBB2*,* TP53*,* ARID1A*, or *EP300* (*p* > 0.05). However, *STK11* showed significantly lower expression in the presence of mutations (*p = 0.0085), Conversely, *FBXW7* showed significantly higher expression in the mutant group (*p = 0.026), suggesting distinct regulatory mechanisms for these two tumor suppressors

Gene expression analysis by mutation status revealed distinct patterns. *KRAS* and *ERBB2* mutations were associated with higher expression levels compared to wild-type (*KRAS*: mean 29.77 vs. 25.10 reads per million (RPM); *ERBB2*: mean 343.26 vs. 312.95 RPM). Conversely, *NOTCH1* (mean 30.32 vs. 34.98 RPM) and *PTEN* (mean 21.68 vs. 24.86 RPM) mutations correlated with reduced expression. *STK11* mutations were associated with significantly lower expression compared to non-mutant (mean 32.34 vs. 47.92 RPM; *p = 0.0085). *TP53* (mean 107.81 vs. 93.27 RPM) and *ARID1A* (mean 44.07 vs. 38.74 RPM) mutations showed modestly higher expression, while *EP300* expression was unchanged (mean 25.23 vs. 25.11 RPM). *FBXW7* mutations were associated with significantly higher expression compared to non-mutant (mean 13.89 vs. 11.97 RPM; *p = 0.026) (Fig. 6). Collectively, these findings indicate that *PIK3CA*, *ERBB2*, and *STK11* mutations are differentially distributed across tumor grade, nodal status, and T stage, respectively.

### IHC analysis of hub genes

**Fig. 7:**
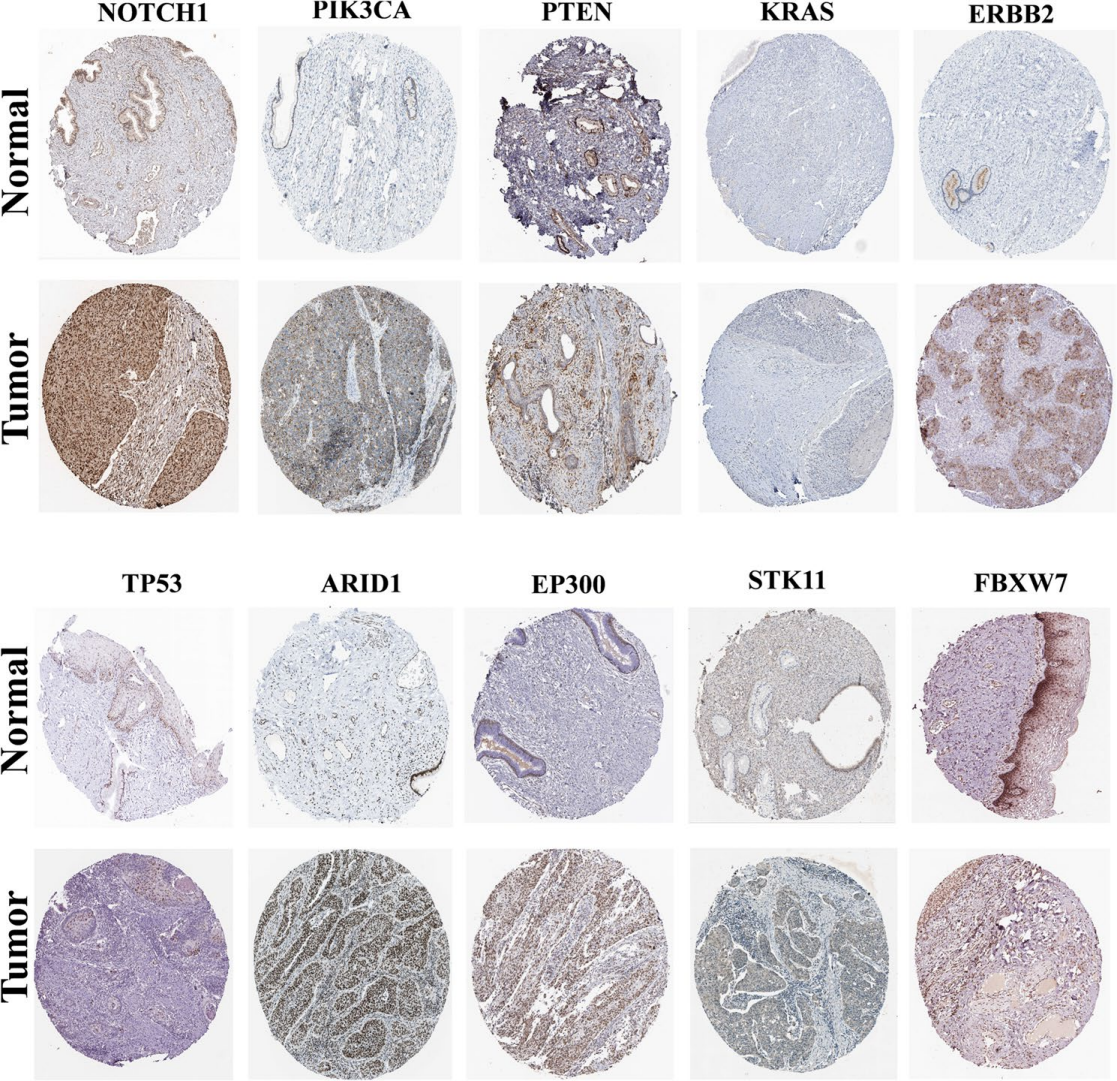
Differential protein expression of hub genes in normal versus cervical cancer tissues analyzed by IHC. IHC analysis of 12 hub driver genes revealed distinct protein expression patterns between normal and cervical cancer samples. PIK3CA, ARID1A, EP300, and NOTCH1 are predominantly expressed at high to medium levels in tumors compared with normal tissues. In contrast, the expression of PTEN, KRAS, TP53, and STK11 is reduced or absent in cancer samples. ERBB2 and FBXW7 are moderately expressed in a subset of cases, indicating heterogeneous protein expression

Immunohistochemical analysis of the 10 hub genes revealed distinct expression patterns across cervical cancer samples (Fig. 7). High or medium protein expression was detected for PIK3CA (8/12 patients, 66.7%), ARID1A (11/12, 91.7%), and EP300 (12/12, 100%) in cancer samples. Conversely, low or no expression was predominant for PTEN (10/12, 83.3%), KRAS (9/10, 90%), TP53 (11/12, 91.6%), and STK11 (9/12, 75%). NOTCH1 was highly/midly expressed in most of the samples (12/12, 100%). ERBB2 (5/12, 41.6%) and FBXW7 (6/12, 50.0%) were moderately expressed. Overall, these findings highlight distinct protein expression profiles between normal and cancer tissues, supporting the functional relevance of these hub genes in cervical cancer pathogenesis.

## Discussion

Cervical cancer can develop because of persistent infection with high-risk types of HPV [7]. Aberrant genomic changes are critical for cervical carcinogenesis and have been shown to impact clinical outcomes [6]. Identifying cancer-associated mutations and understanding their functions are essential for developing personalized therapies. Earlier in silico analyses of cervical cancer relied completely on the TCGA-CESC dataset and focused on mutation frequency alone. In our study, we combined four independent cohorts that included 467 cases originating from diverse geographical regions, enabling us to detect reproducible cross-cohort driver genes. Importantly, we employed a network-based approach to rank the top ten hub genes based on their degree centrality within the PPIN, thus overcoming the limitation of ranking hub genes using mutation frequency alone. Furthermore, we comprehensively predicted potential genotype–immune phenotype relationships by linking hub gene mutations to multiple survival endpoints and immune infiltration patterns. Finally, we performed drug‒gene interaction analysis based on the mutation profiles of the hub genes. Collectively, this integrative framework moves beyond a simple enumeration of driver genes to provide a functionally annotated, clinically contextualized resource for targeted therapy development.

TCGA-CESC is one of the largest comprehensive cervical cancer genomic studies conducted thus far. Previous studies have demonstrated that the reanalysis of publicly available genome-wide datasets can be used to identify genomic and epigenetic aberrations and associated mechanisms related to cervical cancer [23, 24]. In this study, we analyzed 4 cervical cancer genome-wide studies to investigate driver genes and understand their molecular associations via an in silico approach. Whole-genome characterization by TCGA revealed five novel genes whose expression was significantly mutated (SHKBP1-ERBB3-CASP8-HLA-A-TGFBR2) and nine genes whose expression was recurrently mutated (PIK3CA-EP300-FBXW7-HLA-B-PTEN-NFE2L2-ARID1A-KRAS-MAPK1) in patients with cervical carcinomas. Secondary analysis of TCGA cervical cancer data revealed ten genes with the highest mutation burden: TTN, PIK3CA, the mucin family members MUC4/MUC16, the chromatin modifiers KMT2C/KMT2D, and the structural genes SYNE1/FLG/DST, along with EP300 [12]. Driver gene analysis from the same study revealed ten clinically relevant candidates, namely, GPR107 (a GPCR), CHRNA5 (nicotinic receptor), and Rb1 (cell cycle regulator), along with seven additional oncogenic drivers, including DDX3X and H2BFM [12]. However, our analysis revealed 44 driver genes, with *PIK3CA*,* KMT2C*,* KMT2D*,* FBXW7*,* FAT1*,* EP300*,* TP53*,* NOTCH1*,* STK11*, and *CASP8* being the top ten most frequently mutated genes. Furthermore, *PIK3CA*,* NOTCH1*,* PTEN*,* KRAS*,* ERBB2*,* TP53*,* ARID1A*,* EP300*,* STK11*, and *FBXW7* were the top ten hub genes. Our analysis suggested that the number of mutations, their frequencies and driver genes varied among the 4 independent datasets analyzed. Hence, we speculate that while the incidence of gene mutations varies across ethnic backgrounds, many genes that can be used for targeted therapies remain common despite these differences. For example, *PIK3CA* and *STK11* emerged as the most frequently and commonly mutated genes in all four datasets analyzed. Targeting PIK3CA and STK11 or their downstream pathways represents a potential therapeutic strategy that warrants further investigation in cervical cancer [20, 21]. Comparison of mutation with expression using the TCGA-CESC cohort revealed that driver mutations can affect mRNA abundance. The cross-validation of the expression of the hub genes using IHC suggested frequent dysregulation of PI3K/AKT/mTOR pathway components (PIK3CA upregulation and PTEN loss) and chromatin modifiers (EP300/ARID1A upregulation).

GO and pathway analyses help in predicting the functions and pathways of a set of genes in an organism [25]. We performed pathway enrichment and GO analyses to understand the biological functions and pathways affected by driver genes. We noted that pathways associated with viral pathogenesis were significantly enriched, as were pathways related to hepatocellular carcinoma, prostate cancer, and endometrial cancer. Many of these pathways are known to promote cervical cancer. GO term analysis revealed enrichment of kinase activity-related genes. Furthermore, kinase activity activation has been shown previously to promote cancer development and therapeutic resistance in patients with cervical cancer [26].

The PPIN approach is used by researchers to identify interactions between proteins, predict their functions, and identify highly connected genes or hub genes [13]. We employed the PPIN and CytoHubba approaches to functionally prioritize hub genes. This network-based approach identifies genes that are most functionally impactful when mutated. Following the results of the GO and pathway analyses, we generated a PPIN of 44 driver genes to identify the top ten highly connected genes. This analysis suggested that the top 10 hub genes (*PIK3CA*,* NOTCH1*,* PTEN*,* KRAS*,* ERBB2*,* TP53*,* ARID1A*,* EP300*,* STK11*, and *FBXW7*) occupy the central topological position of the PPIN [12, 14, 27]. Furthermore, our study uniquely combines the associations of the top 10 hub genes with survival, cancer hallmarks, immune infiltration, and drug–gene interactions in patients with cervical cancer. This study demonstrated that altered hub gene expression contributes to the establishment of fundamental cancer hallmarks, potentially driving multiple aspects of carcinogenesis.

We performed multivariate analysis to understand the impact of the single-nucleotide variation burden on cervical cancer patient survival. Cervical cancer patients with mutations in driver hub genes presented shorter overall survival, progression-free survival, disease-specific survival, and relapse-free survival. The imbalance in sample size between the high- and low-risk groups is attributable to the low prevalence of mutations in the selected hub genes. Therefore, the findings of the present study require validation in independent cohorts of samples with larger sample sizes. Our analysis revealed that *PIK3CA* was the most frequently mutated gene. Studies have reported that PIK3CA activation can promote tumor growth by increasing proliferation, migration, and cell survival [28–30]. A correlation between poor treatment response and low survival rates with mutations in *PIK3CA* has already been reported [29, 30]. Previous studies have reported PTEN loss in multiple cancers, including cervical cancer [31]. Harima and colleagues demonstrated that cervical cancer patients with PTEN mutations exhibit accelerated tumor progression and reduced radiosensitivity, leading to poor therapeutic outcomes [32]. *PTEN* mutations are associated with poor survival in patients from China [33]. The activation of KRAS by gain-of-function mutation and its contribution to cervical cancer have been well reported [34]. Compared with patients in the wild-type cervical cancer patient cohort in China, patients with *KRAS* mutations have poorer relapse-free survival [34]. *ERBB2*, commonly known as *HER2*, is a well-established oncogene. Gain-of-function mutations in *ERBB2* have been reported in patients with cervical cancer and have been identified as promising targets in patients with this disease [35]. High-risk HPV types promote cervical malignancy partly through the ability of their E6 oncoproteins to downregulate TP53 expression, effectively disabling this crucial tumor suppressor pathway [36]. Patients with *TP53* mutations have poor survival rates among patients with cervical cancer [37]. Molecular studies have established the tumor suppressor function of ARID1A via its role in the SWI/SNF chromatin remodeling machinery, which orchestrates transcriptional programs that control cellular growth, differentiation, and DNA repair fidelity [38]. A reduction in ARID1A expression contributes to reduced overall survival in patients with cervical cancer [39]. EP300 is a histone acetyltransferase that is critical for controlling proliferation and differentiation [40]. A study by Yoshimoto and coworkers revealed recurrent EP300 mutations in 12.2% of a cervical cancer patient cohort from Japan [41]. EP300 mutation contributes to poor prognosis in lymph node-positive cervical cancer patients from China [42]. *STK11* demonstrates tumor suppressive activity because of its dual role in maintaining proper cell polarization and governing metabolic pathways [43]. The genomic alteration of *STK11* contributes to poor prognosis and promotes cervical cancer [44]. Peutz–Jeghers syndrome patients with germline mutations in *STK11* presented an increased risk for the development of cervical cancer [45]. Earlier studies reported that *FBXW7* is a tumor suppressor gene [46]. FBXW7 promotes apoptosis by targeting EMT and RhoA signaling [47]. *FBXW7* mutations promote proliferation, migration, and invasion in cervical cancer [48]. While our survival analysis identified candidate prognostic driver genes within the TCGA, these findings have not been externally validated in independent, prospective cohorts. Therefore, the prognostic performance reported here may be overestimated, and further validation is needed before clinical application. Despite this, the association between mutation and patient survival strengthens the clinical relevance of these driver genes.

We performed an investigation to predict the associations between driver hub genes and tumor immune infiltration, as these cells are key components of the tumor microenvironment and have the potential to influence tumor growth and immune evasion [49]. Our analysis suggests the potential role of genomic alteration in immune infiltration and the tumor microenvironment. We found statistically significant associations between driver hub gene mutations and altered immune cell infiltration patterns, particularly those affecting TH2 cells, natural killer (NK) cells, dendritic cells (DCs), and γδ T cells. TH2 cell infiltration has been reported earlier, with poor survival, recurrence, and metastasis in patients with malignancies [50]. TH2-mediated inflammation plays a role in the development of cervical cancer from HPV-positive cervical cells [51]. DCs play crucial roles in generating antitumour immune responses to fight against cancer [52]. However, findings from recent studies have suggested a potential role for DC infiltration in driving metastasis through the establishment of immune suppression. Hence, DCs, while essential for antigen presentation and the initiation of anti-suit responses, may exhibit dysfunctional phenotypes within the tumor microenvironment, thereby promoting immune escape. Similarly, gamma delta T-cell infiltration plays both tumor-promoting and tumor-inhibitory roles [53]. A previous study reported that gamma delta T cells increase immunotherapy efficacy in patients with cervical cancer [54]. Immune infiltration analysis revealed a statistically significant difference in cell type abundance across different sample groups. Our findings are based on statistical analysis of in silico data and do not reveal causal relationships between specific genomic alterations and immune landscape remodeling. The MutSigCV and OncoKB tools were used to map the immune infiltration profile with known driver genes to understand the biological context. Gamma_delta cell infiltration correlated with extreme driver mutation, whereas TH2_NK and DC infiltration correlated with lower driver mutation. Our analysis revealed a significant association between mutation burden and immune cell infiltration patterns, which may be relevant to immune evasion. Although correlated, the findings of the present study align with the observation that missense mutations in genes such as TP53 and KRAS can modulate the tumor microenvironment via altered chemokine secretion and antigen presentation machinery. Thus, driver hub gene mutations are statistically correlated with alterations in both tumor-intrinsic pathways and the immune landscape. However, further experimental studies are needed to determine their functional impact on disease progression and therapeutic response.

The immune infiltration findings presented in the present study are correlative and do not suggest causation. Our analysis revealed significant associations between specific driver gene mutations and the abundance of certain immune cell types (e.g., TH2, NK, DC, and γδ T cells). However, whether these mutations directly remodel the immune landscape or vice versa require experimental validation. Furthermore, the use of algorithms such as CIBERSORT and ImmuCellAI provides estimates rather than direct measurements of immune cell abundance. Therefore, these hypothesis-generating observations require experimental validation using multiplex immunohistochemistry, flow cytometry, or single-cell RNA sequencing in independent patient cohorts.

Finally, we conducted a drug‒gene network analysis to identify potential therapeutic compounds that can be tested in specific genetic backgrounds. Previous studies have shown that drug‒gene interactions have helped researchers identify druggable genes from genome-wide studies. Among the hub genes, genes such as *STK11*,* NOTCH1*,* PIK3CA*,* FBXW7*,* ERBB2*,* PTEN*,* EP300*,* KRAS*, and *TP53* were identified as druggable candidates via PanDrug2 analysis. Our study identified approved drugs as well as drugs under clinical trials. This study also revealed that PI3K inhibitors, EGFR inhibitors, mTOR inhibitors, VEGFR inhibitors, AKT inhibitors, PDGFR tyrosine kinase receptor inhibitors, tubulin polymerization inhibitors, and topoisomerase inhibitors may be useful for managing cervical cancer patients. It is critical to emphasize that these drug–gene predictions are purely in silico. It is critical to note that these drug–gene predictions are computational and require preclinical and clinical validation before translation. Our investigation has several novelties compared with previous driver gene analyses in cervical cancer. First, while earlier studies identified frequently mutated genes, we used a network-based approach to identify genes that may be critical in cervical cancer. Second, we analyzed four independent cohorts of patients with cervical cancer to identify genes that are commonly affected. Third, we linked mutation status not only to overall survival but also to progression-free, disease-specific, and relapse-free survival, providing a more complete prognostic picture. Fourth, we stratified mutations by functional class (missense driver, truncating driver, and VUS) to determine whether missense drivers of *PIK3CA*, *ERBB2*, and *TP53* are associated with elevated mRNA expression compared with wild-type or truncating mutations, a finding with potential therapeutic implications. Fifth, we performed IHC-based protein validation for all the hub genes. Sixth, a systematic approach used for drug–gene interactions identified a prioritized list of compounds and drug families that can be useful for preclinical testing. Thus, while individual driver genes are not entirely novel, the integrative, multicohort, network-prioritized, mutation-type-resolved, and multi-endpoint survival framework represents a novel approach.

## Limitations

Although our study is comprehensive, we acknowledge several limitations. (1) This study analyzed publicly available cervical cancer genomic datasets via online tools. (2) The complete observation relied on in silico analysis without any validation or functional studies. (3) High-risk HPV infection is a critical risk factor for CC. However, the association between HPV types and hub gene alterations could not be analyzed because of a lack of HPV genotyping information in all the samples. Future studies that combine HPV genotyping with multiomics data may offer better insights into specific molecular mechanisms and enhance clinical relevance. (4) The immune infiltration analyses are completely correlational. The observed associations between driver gene mutations and immune cell abundances do not establish directionality or causality. (5) Hub gene identification by CytoHubba is purely topological. Hence, all network-derived conclusions require experimental validation. Owing to the purely in silico nature of this study, all the findings, including driver gene predictions, survival associations, and drug‒gene interactions, are correlative and hypothesis-generating. Therefore, all conclusions require experimental validation through in vitro and in vivo models before clinical translation. Despite these limitations, we performed extensive in silico investigations and used data from 4 different datasets to identify driver genes and their possible role in cervical cancer. Future experimental validation, including functional studies and independent cohort analyses, will be necessary to confirm these in silico observations.

## Conclusions

In this study, driver genes statistically associated with cervical squamous cell carcinoma were identified via in silico analysis of publicly available datasets. Overall, our study identified 44 recurrently mutated genes in cervical SCC, along with their associated genetic alterations and dysregulated biological pathways. Integrative multiomics analysis reinforced known driver mutations and generated hypotheses regarding their collective impact on prognosis, immune infiltration, and potential therapeutic targeting. The novelty of the present study is the use of integrative, multicohort, and network-based approaches to prioritize which driver genes are most functionally impactful and therapeutically relevant using an in silico method. While our study provides a prioritized list of candidate driver genes, experimental validation using in vitro and in vivo models is essential for confirming their functional significance and therapeutic potential.

## Supplementary Information

Supplementary Material 1: Supplementary Table 1: List of driver genes identified and their mutation frequency.

Supplementary Material 2: Supplementary Table 2: Driver hub genes and cancer hallmark gene set analysis.

Supplementary Material 3: Supplementary Table 3: Drive hub gene mutation and immune infiltration analysis.

Supplementary Material 4: Supplementary Table 4: Drug–gene interaction analysis.

Supplementary Material 5: Supplementary Table 5: Clinically prioritized drug–hub gene interactions in cervical squamous cell carcinoma identified through integrative in silico analysis.

Supplementary Material 6: Supplementary Table 6: Mutation frequency & expression by clinical parameters.

## Data Availability

The datasets used and/or analyzed during the current study are available from the corresponding author on reasonable request.
